# Emergency Stress, Hardiness, Coping Strategies and Burnout in Health Care and Emergency Response Workers During the COVID-19 Pandemic

**DOI:** 10.3389/fpsyg.2022.918788

**Published:** 2022-06-21

**Authors:** Monia Vagni, Tiziana Maiorano, Valeria Giostra, Daniela Pajardi, Paul Bartone

**Affiliations:** ^1^Department of Humanities, University of Urbino, Urbino, Italy; ^2^Institute for National Strategic Studies, National Defense University, Washington, DC, United States

**Keywords:** hardiness, Emergency Stress, Burnout, COVID, health care

## Abstract

**Objective:**

Stress is a growing problem in the general population, but most especially for workers responding to the COVID-19 crisis. The present study examines stress and Burnout in Health Care workers and Emergency Responders during the third COVID wave in Italy. In addition, we explore the value of psychological Hardiness and positive coping strategies as protective factors against the ill-effects of stress.

**Methods:**

Over a 5-month period in 2021, surveys were administered across all Italian regions to several groups including Health Care workers (*N* = 220), Emergency Responders (firefighters, civil protection, ambulance personnel; *N* = 121), volunteer Italian Red Cross workers (*N* = 328), and a comparison group (*N* = 400) drawn from the General Population of Italy.

**Results:**

Results showed that among the groups, Health Care workers had the highest levels of Emergency Stress, while the Red Cross volunteers had relatively lower stress levels. Hardiness and positive coping levels were highest among Red Cross workers, and lowest in the General Population sample. The biggest impact on Burnout was seen among health care workers, especially on Emotional Exhaustion and Depersonalization. Multiple regression results showed that Hardiness operates as a moderator of the effects of Emergency Stress on Emotional Exhaustion and Depersonalization aspects of Burnout, while problem focused coping and Stopping Negative Thoughts-Emotions also showed moderating effects.

**Conclusion:**

These results suggest that Health Care workers and Emergency Responders would benefit from additional training in hardiness and positive coping skills.

## Introduction

The confrontation with stressful events such as the COVID pandemic poses a threat to psychological health and wellbeing for the general population, but an even greater risk for Health Care workers who face added stressors related to the health emergency. Several studies have attempted to identify factors that provide resilience for Health Care workers in the COVID pandemic (Croghan et al., 2021; Hines et al., 2021). These studies have tended to focus on social and demographic factors that may increase resilience or vulnerability (e.g., social support, age, gender). Few studies to date have examined personality and coping dimensions that may add to resilience for Health Care and Emergency workers responding to the COVID crisis (Conversano et al., 2020; Maiorano et al., 2020).

Previous studies have identified positive coping strategies and hardiness as buffers against the negative effects of stress in general (Kobasa, 1982; Kobasa et al., 1982). Hardiness, defined by Kobasa (1979) as a personality trait consisting of three fundamental characteristics (commitment, control and challenge), is a protective factor against the negative effects of stress including burnout (White et al., 2020; Bartone et al., 2021). Several recent studies have also found that this psychological resource is a significant buffer against the adverse psychological effects of stress related to the COVID pandemic, for Health Care and Emergency workers (Maiorano et al., 2020; Vagni et al., 2020a) and also in the general population (Dymecka et al., 2021). Furthermore, hardiness is a predictor of adaptive and positive coping (Clarke, 1995; Lease, 1999; Chan, 2000; Bartone and Homish, 2020; Kamtsios and Bartone, 2021). Higher hardiness levels are also associated with less use of avoidance coping in both military and civilian groups (Thomassen et al., 2018, 2022). Vagni et al. (2020b) recently examined emergency volunteers from the Red Cross, and found that hardiness showed significant effects in reducing emergency stress levels, emotional exhaustion, and depersonalization, and maintaining a sense of personal accomplishment. Hardiness has also shown benefits among the general population, facilitating perceptions of the pandemic as a challenge (Dymecka et al., 2021).

According to recent work by Stein and Bartone (2020), a hardiness mindset can be increased in various ways, including as a function of successful coping with various threats and challenges. For example Vagni et al. (2021) studied Health Care and Emergency workers during the COVID pandemic, and found that workers' hardiness skills increased from the first to the second wave of the pandemic, while their coping strategies remained fairly stable. Similar findings were reported by Labrague (2021). These results are especially important because they show that in situations in which emergency stress is prolonged, hardiness may be an effective protective factor against the negative effects of stress such as burnout.

During stressful life events, as is happening now in the long pandemic period, hardiness may influence mental health through increasing positive, active coping approaches and decreasing the use of dysfunctional coping strategies (Thomassen et al., 2022). According to Bartone et al. (2022) people who are higher in hardiness tend to use more adaptive coping styles that prompt them to take actions to remove the sources of stress. In a meta-analytic review, Eschleman et al. (2010) also found that hardiness was linked to more active, problem solving coping styles. Therefore, it was expected that higher levels of hardiness would be related to more adaptive coping styles.

## Objectives

Hardiness is a protective factor against the negative effects of the COVID pandemic stress both for the General Population and for Health Care and Emergency Responders (Maiorano et al., 2020; Dymecka et al., 2021; Manchia et al., 2022; Vagni et al., 2021). Hardiness has a strong association with positive coping strategies both in the General Population and in Health Care and Emergency Responders (Bartone and Homish, 2020; Kamtsios and Bartone, 2021; Vagni et al., 2021).

Previous studies have verified the positive effects of Hardiness and positive coping strategies on the stress associated with the COVID-19 considering only the General Population or first responders. The general objective of this study is to verify whether the coping and Hardiness skills used by the General Population to cope with COVID-19 are the same as those used by Health Care and Emergency Responders (first study). In fact, the main hypothesis is that Health Care and Emergency Responders need a greater capacity for resilience and different coping strategies. The results obtained from this first study led to consider the group of the General Population too different to continue in subsequent analyzes to keep it together with Health Care and Emergency Responders.

A second general hypothesis is that Volunteers Emergency Responders show different levels of Hardiness and positive coping from Health Care and professional Emergency Responders.

The first study examined possible differences between the General Population and Health Care Workers (HCWs), as well as both professional Emergency Responders (ERs) and Volunteer Emergency Responders (VERs) in terms of coping and resilience skills. In the second study involving HCWs, ERs and VERs, the protective effects of Hardiness and coping strategies with respect to Burnout were evaluated, as well as their potential role as moderators in the stress—illness relation. As seen in previous studies (Vagni et al., 2020b, 2021), the major stress experienced by Health Care and Emergency Response personnel is linked to specific factors in their work environment, and is not generic. This led to the choice of instruments for measuring the different stressors between HCWs, ERs, VERs and the General Population.

### Main Hypotheses

#### Study 1

1. Health Care Workers, Professional Emergency Responders, and Volunteer Emergency Responders have different coping strategies and resilience skills compared to the General Population.
2. In the General Population, Hardiness and coping strategies play a protective role regarding perceived stress and stress reactions during the COVID-19 pandemic.

#### Study 2

1. Health Care Workers experience higher levels of Emergency Stress and Burnout than both professional and Volunteer Emergency Responders.
2. Hardiness and positive Coping strategies reduce the risk of developing work-related stress problems in operators involved in the management of the pandemic.
3. Hardiness and coping strategies serve as moderators lowering the impact of Emergency Stress on Burnout.

## Participants

Subjects recruited for these studies were as follows:

- *N* = 400 participants from the General Population (GP) with a mean age of 31.95 (SD = 13.21; min–max = 18–87). The General Population group included 312 females (78%) and 88 males.
- *N* = 220 Health Care Workers (HCWs) with a mean age of 43.80 (SD = 12.13; min–max = 20–68) and included: 99 (45%) physicians and 121 (55%) nurses. Of the HCWs, 21.8% were males and 66.9% females. Of these, 60.5% were frontline workers dealing with COVID-19 patients.
- *N* = 121 Emergency Responders (ERs) included firefighters, civil protection, and ambulance personnel, with a mean age of 47.53 (SD = 11.99; min–max = 20–73). Of these, 53.7% were male and 46.3% female, and 54.5% were frontline workers with COVID-19 patients.
- *N* = 328 Volunteer Emergency Responders of the Italian Red Cross (VERs) with a mean age of 47.0 (SD = 14.52; min–max = 18–77). Of these, 40.2% were male and 59.8% female, with 50.9% of the VERs performing direct interventions on COVID-19 patients.

Any participant who treated COVID-19 patients directly was further classified as a “COVID patient.”

### Procedure

Both studies used an online transactional survey, and participants were recruited from May to September 2021 from all Italian regions. The survey included online informed consent, several questionnaires and basic sociodemographic information. Participants' anonymity was maintained while collecting the data. All procedures used in both studies were approved by the Ethics Committee of the University of Urbino (Comitato Etico per la Sperimentazione Umana—CESU). The research was conducted in compliance with the ethical principles of research established in the “Declaration of Helsinki” and in the “Convention on Human Rights and Biomedicine” (Oviedo Convention).

### Materials

Some scales were administered to all participants of the four groups, such as those to measure Hardiness and coping strategies. Other scales were differentiated for the General Population. The HCWs, ERs and VERs received the same test battery, since both were directly involved in responding to the pandemic emergency; their measures included Emergency Stress and risk of developing Burnout from work-related stress.

- The Hardiness Resilience Gauge (HRG) is a 28-item scale that measures total hardiness as well as the three hardiness facets of commitment, control and challenge (Bartone et al., 2022). Reported Cronbach's alpha reliability coefficients are high, at 0.93 for total hardiness, and 0.85, 0.84, and 0.89 for commitment, control and challenge respectively. Sample items are “I look forward to my daily activities” (commitment), “I am responsible for my own success in life” (control), and “I find the positives in any life change” (challenge). The Italian version of the HRG was created following a simple back translation method: four Italian researchers and psychologists translated the original version into Italian and a native English speaker, professor of scientific English in psychology, re-translated the scale into English, without knowing the original version. A comparison was made between reverse translation and the original version and two items were discussed that have minimal differences from the original English version, to refine the Italian version. In the present reference sample (General Population) the HRG also showed good reliability, with Cronbach's alpha coefficients for the total scale (α = 0.91) and for the three facets: Commitment (α = 0.86); Challenge (α = 0.82) and Control (α = 0.80).
- The Coping Self-Efficacy Scale—Short Form (CSES-SF, Chesney et al., 2006) is a 13-item self-report questionnaire that evaluates perceived self-efficacy for coping with challenges and threats. The instrument is composed of three sub-scales: Problem-Focused coping (for example, “Make an action plan and follow it when faced with a problem”), Stopping Negative Thoughts-Emotions (“Keep your mind away from negative thoughts”), and Support (“Seek moral support from friends and family”). The subject was asked to rate on an 11-point scale the degree to which they believed they could adopt important behaviors for adaptive coping on an eleven-point Likert scale, with scores ranging from 0 (cannot do at all) to 10 (certain can do). It has previously been validated and found effective in measuring coping strategies in Health Care and emergency workers during the COVID-19 pandemic (Chesney et al., 2006; Vagni et al., 2020b, 2021).

The following was administered to the General Population only:

- The Perceived Stress Scale (PSS) is a 10-item measure of perceived stress in life (Cohen et al., 1983; Cohen and Williamson, 1988). The items assess to what degree a person feels his life is overloaded, unpredictable, or uncontrollable. The scale also contains a series of direct questions about reactions to stress (for example: “In the last month, how often have you been upset because of something that happened unexpectedly?” and “In the last month, how often have you felt nervous and stressed?”). The PSS was designed for use in samples of the general population with an educational level at least equal to lower middle school. The items and the alternatives for the answer are easy to understand. For each item, respondents are asked to indicate how often they felt a certain way (range 0–4). In the present study, the PSS was administered only to the General Population sample.

The following tools were administered to the Health Care, Emergency Responders, and Volunteer Emergency Responders:

- Emergency Stress Questionnaire (ESQ, Maiorano et al., 2020; Vagni et al., 2020a,b,c,d): The ESQ is a self-report instrument, already published and validated in previous research, to assess several level of Emergency Stress: organizational relational, physical, inefficacy decisional, emotional, cognitive, and COVID-19 stress. The ESQ consists of 33 items assessed on a five-point Likert scale, with scores ranging from 0 (not at all) to 4 (very much). The Cronbach reliability coefficients for the individual scales relating to this sample are all satisfactory: Total ESQ (α = 0.93), Organizational-Relational stress (α = 0.71), Physical stress (α = 0.87), Inefficacy Decisional stress (α = 0.75), Emotional stress (α = 0.78), Cognitive stress (α = 0.67) and COVID-19 stress (α = 0.76).
- Maslach Burnout Inventory—Human Services survey—Italian version (MBI—HSS, Maslach and Jackson, 1986; Sirigatti and Stefanile, 1993; Loera et al., 2014): This is a self-report questionnaire and a specific version to measure the presence of burnout in Health Care workers. The Italian version of MBI—HSS has 20 self-scored items on a seven-point frequency scale ranging from 0 (never) to 6 (every day) and has three subscales, as follow: Emotional Exhaustion (EE), Depersonalization (D), and Personal Accomplishment (PA). Emotionally Exhausted (EE) employees lack adaptive resources and feel that emotional resources are so depleted that they cannot give any more to their jobs. Depersonalization (D) refers to impersonal, negative, and indifferent responses to the care and treatment to be provided to patients. Finally, Personal Accomplishment (PA) refers to a sense of self-efficacy, a feeling of competence as well as a tendency to evaluate oneself positively, and low scores in this scale correspond to higher degrees of experienced burnout. The PA scale is completely independent of the other two scales (Maslach and Jackson, 1981; Maslach et al., 1996).

### Statistical Strategy

#### Study 1

To test for differences in coping strategies and Hardiness skills between the HCWs, ERs, VERs and General Population Groups, a one-way ANOVA was conducted with Bonferroni's *post-hoc* comparisons (Hy1).

To test for effects of hardiness and coping strategies in the General Population, a linear regression model was generated with PSS as the dependent variable, and age, gender, HRG and CSES-SF scales as predictors (Hy2).

#### Study 2

To test for the presence of higher levels of Emergency Stress and a higher risk of developing work-related stress problems in Health Care Workers and both Emergency Responders and Volunteers Emergency Responders, a one-way ANOVA was performed comparing the three groups (Hy3). To test for the protective effect of resilience capacities (HRG) and coping strategies (CSES-SF) on Burnout levels (MBI-HSS), Pearson correlations were first calculated to identify associations among the variables of interest, and then three distinct multiple regression models (H4) were generated.

Finally, Hardiness and coping were investigated as potential moderators of the impact of Emergency Stress (ESQ) on Burnout, applying a series of OLS regression models including appropriate interaction terms (Hy5). Interaction effects are displayed graphically using procedures contained in the PROCESS macro (version 4.0) for SPSS (Hayes, 2022).

## Results

### Study 1

Using *t*-tests to compare means, physicians and nurses were found not to differ on any of the variables except for Total ESQ scores, on which nurses showed higher stress levels (*t* = −4.197; *p* < 0.001). Thus, physicians and nurses were combined into a single Health Care Worker (HCW) group.

In addressing hypothesis 1, a series of one-way ANOVAs were conducted with Bonferroni's *post-hoc* comparisons. These results are displayed in Table 1. Health Care, and both professional and volunteers Emergency Responders showed more positive coping strategies and Hardiness skills than the General Population.

**Table 1:** One way ANOVAs between HCWS, ERs, VERs and GP on HRG and CSES-SF scores.

	**HCWs (*N* = 220)**	**ERs (*N* = 121)**	**VERs (*N* = 328)**	**GP (*N* = 400)**	***F***	***Post*−*hoc*^a^**
	**Mean (SD)**	**Mean (SD)**	**Mean (SD)**	**Mean (SD)**		
**HRG**						
Commitment	22.19 (4.58)	23.05 (4.44)	24.53 (4.22)	20.46 (5.34)	44.73^**^	H – GP = 1.73^**^
						ER – GP = 2.59^**^
						VER – GP = 4.07^**^
						VER – H = 3.34^**^
						VER – ER = 1.48^*^
Control	17.10 (3.80)	17.13 (3.57)	17.44 (3.49)	16.95 (4.01)	1.06	
Challenge	21.40 (4.61)	22.45 (4.29)	23.72 (3.94)	20.01 (5.02)	41.59^**^	H – GP = 1.40^**^
						ER – GP = 2.44^**^
						VER – GP = 3.72^**^
						VER – ER = 1.28^*^
						VER – H = 3.2 2^**^
Tot HRG	60.70(10.70)	62.63(10.44)	65.69 (9.82)	57.42(11.98)	35.38^**^	H – GP = 3.28^**^
						ER – GP = 5.21^**^
						VER – GP = 8.28^**^
						VER – H = 5.00^**^
						VER – ER = 3.06^*^
**CSES-SF**						
Problem Focused	38.40 (6.72)	39.59 (86.86)	39.39 (6.81)	35.45 (7.60)	22.20^**^	H – GP = 1.95^*^
						ER – GP = 4.13^**^
						VER – GP = 3.94^**^
						VER – H = 1.99^*^
						H – ER = −2.19^*^
Stop Negative T-E	31.70 (10.71)	37.16 (9.83)	36.79 (10.74)	29.17 (11.62)	36.05^**^	H – GP = 2.53^*^
						ER – GP = 7.99^**^
						VER – GP = 7.62^**^
						VER – H = 5.09^**^
						H – ER = −5.46^*^
Support	20.06 (7.29)	21.45 (6.32)	21.65 (6.86)	21.16 (5.46)	2.88^*^	ER – H = 1.39^*^
						VER – H = 1.59^*^

*HCWs or H, Health Care workers; ERs, Emergency Response Personnel; VERs, Volunteer Emergency Response; GP, General Population; HRG, Hardiness Resilience Gauge; CSES-SF, Coping Self-Efficacy Scale—Short Form*.

a *Only significant comparations are included*.

* *p ≤ 0.05*;

** *p ≤ 0.001*.

The highest Hardiness levels were seen in the Volunteer Emergency Responder (VER) group (Mean = 65.69, SD = 9.82), and the lowest levels in the General Population (GP) sample (Mean = 57.42, SD = 11.98). Similarly, the GP group showed the lowest levels of Problem Focused and Stopping Negative Thoughts-Emotions coping approaches, while Emergency Responders were comparatively high in these positive coping strategies. In terms of Support coping, the only difference observed was with Health Care Workers, show showed lower Support Seeking than Emergency Responders.

#### Hypothesis 2

To test for a protective role of HRG and CSES-SF scales on stress in the General Population sample, a linear regression model was generated, controlling for age and gender and predicting scores on the Perceived Stress Scale (PSS). The model was significant, explaining 25% of the variance (*R*^2^ = 0.253; *F* = 16.279; *p* < 0.001) in PSS scores. Significant predictors were Hardiness Challenge (Beta = −0.125; *p* < 0.05), Problem Focused coping (Beta = −0.125; *p* < 0.05) and Stopping Negative Thoughts-Emotions (Beta = −0.165; *p* < 0.01) coping. Lower scores on all these predictors was associated with higher levels of reported stress. Younger age (Beta = −0.180; *p* < 0.001) and female gender (Beta = 0.180; *p* < 0.001) were also significant predictors, and thus appear to be risk factors for increased stress reactions in the General Population.

#### Hypothesis 3

To test if there are higher levels of Emergency Stress and Burnout among Health Care Workers, a one-way ANOVAs were performed contrasting the HCWs, ERs and VERs groups on these variables. Comparisons between groups were made with Bonferroni *post-hoc* test (Table 2). Results indicate that Health Care Workers are experiencing the highest levels of Emergency Stress and Burnout (Emotional Exhaustion, Depersonalization), significantly more than both Professional and Volunteer Emergency Responders, Hypothesis 4 posits that Hardiness skills and coping strategies exert a protective effect against Burnout, and allow for a greater sense of Personal Accomplishment. In order to test for this, Pearson correlations between the variables of interest were first calculated for the total group of Health Care Workers, Emergency Responders and Volunteer Emergency Responders. Hardiness was seen to correlate substantially with all three coping strategies, and with the three burnout scales. Emergency Stress scores also showed significant correlations with the three burnout scales and (negatively) with the coping scales. Complete correlation results are in Table 3.

**Table 2:** One-way ANOVAs on ESQ and burnout (MBI-HSS) between HCWs (*N* = 220), ERs (*N* = 121) and VERs (*N* = 328).

	**HCWs (*n* = 220)**	**ERs (*n* = 121)**	**VERs (*n* = 328)**	***F***	***Post*−*hoc*^a^**
	**Mean (SD)**	**Mean (SD)**	**Mean (SD)**		
**ESQ**
Organizzational-Relational	18.26 (4.98)	14.66 (5.74)	12.49 (5.09)	81.94^***^	HCW – ER = 3.60^***^
					HCW – VER = 5.77^***^
					ER – VER = 2.17^***^
Physical	11.54 (5.69)	6.98 (5.13)	6.52 (5.15)	62.42^***^	HCW – ER = 4.55^***^
					HCW – VER = 5.01^***^
Inefficacy decisional	12.56 (4.01)	10.30 (4.48)	9.56 (4.06)	35.62^***^	HCW – ER = 2.67^***^
					HCW – VER = 3.01^***^
Emotional	12.80 (4.68)	8.95 (4.81)	8.53 (4.49)	60.14^***^	HCW – ER = 3.85^***^
					HCW – VER = 4.27^***^
Cognitive	7.32 (3.09)	5.01 (2.92)	4.89 (3.00)	46.62^***^	HCW – ER = 2.31^***^
					HCW – VER = 2.43^***^
COVID-19	12.75 (4.52)	10.81 (4.83)	9.53 (4.54)	32.51^***^	HCW – VER = 3.22^***^
					ER – VER = 1.28^*^
					HCW – ER = 18.52^***^
Tot ESQ	75.23 (20.64)	56.71 (22.17)	51.52 (20.18)	88.54^***^	HCW – ER = 18.52^***^
					HCW – VER = 23.71^***^
**MBI-HSS**
EE	23.24 (10.93)	13.31 (8.46)	11.24 (7.76)	120.69^***^	HCW – ER = 9.93^***^
					HCW – VER = 12.00^***^
D	7.49 (6.53)	3.66 (4.89)	3.71 (4.13)	39.74^***^	HCW – ER = 3.83^***^
					HCW – VER = 3.78^***^
PA	26.07 (5.76)	26.88 (5.76)	26.54 (5.46)	1.25	

*HCWs, Health Care workers; ER, Emergency Response; VERs, Volunteer Emergency Responders; ESQ, Emergency Stress Questionnaire; MBI-HSS, Maslach Burnout Inventory—Human Services Survey; EE, emotional exhaustion; D, depersonalization; PA, personal accomplishment*.

a *Only significant comparations are included*.

* *p < 0.05*;

*** *p ≤ 0.001*.

**Table 3:** Pearson correlations between ESQ, HRG, CSES-SF and burnout MBI-HSS scores (*N* = 676).

	**HRG**			**HRG**	**CSES-SF**			**ESQ**
	**Commitment**	**Control**	**Challenge**	**Total**	**PF**	**SNT-E**	**S**	**Total**
**HRG**
Commitment	1	0.54^***^	0.61^***^	0.87^***^	0.50^***^	0.58^***^	0.45^***^	−0.32^***^
Control	0.54^***^	1	0.50^***^	0.79^***^	0.41^***^	0.41^***^	0.30^***^	−0.16^***^
Challenge	0.61^***^	0.50^***^	1	0.85^***^	0.52^***^	0.53^***^	0.36^***^	−0.26^***^
**CSES-SF**
PF	0.50^***^	0.41^***^	0.52^***^	0.58^***^	1	0.65^***^	0.41^***^	−0.20^***^
SNT-E	0.58^***^	0.41^***^	0.53^***^	0.61^***^	0.65^***^	1	0.52^***^	−0.35^***^
S	0.45^***^	0.30^***^	−0.36^***^	0.45^***^	0.41^***^	0.52^***^	1	−0.21^***^
MBI-HSS
EE	−0.35^***^	−0.16^***^	−0.24^***^	−0.30^***^	−0.19^***^	−35^***^	−0.23^***^	0.72^***^
D	−0.32^***^	−0.20^***^	−0.21^***^	−0.30^***^	−0.20^***^	−0.22^***^	−0.20^***^	0.49^***^
PA	0.36^***^	0.36^***^	0.42^***^	0.45^***^	0.40^***^	0.34^***^	0.24^***^	−0.14^***^

*ESQ, Emergency Stress Questionnaire; HRG, Hardiness Resilience Gauge; CSES-SF, Coping Self-Efficacy Scale—Short Form; PF, Problem Focused; SNT-E, Stop Negative Thoughts-Emotions; S, support; MBI-HSS, Maslach Burnout Inventory-Human Services Survey; EE, Emotional Exhaustion; D, Depersonalization; PA, Personal Accomplishment*.

*** *p ≤ 0.001*.

Following this, three separate hierarchical multiple regression models were generated, one for each burnout scale. In consideration of the fact that all ESQ scales show consistently higher levels in the HCWs group compared to the ERs and VERs groups, only total ESQ scores were included in the following analyses. The models included the Burnout scales as dependent variables, and with age, gender, group (HCWs; ERs; and VERs), having managed COVID-19 patients, and HRG scales as predictors in step 1. Coping strategies were added in step 2.

As can be seen in Table 4, the final models were all significant, predicting Burnout Emotional Exhaustion, Depersonalization, and Personal Accomplishment. For Group effects, Health Care Workers are experincing more Burnout (EE and D) compared to Professional Emergency Responders, while the Volunteer Emergency Responders report somewhat less. Younger age is predictive of more Depersonalization Burnout. For the Personal Accomplishment Burnout dimension, Hardiness Control and Challenge, and Problem Focused coping all are significant predictors.

**Table 4:** Hierarchical linear regression models on burnout BMI-HSS scales (*n* = 676).

	**EE**		**D**		**PA**	
	***B***	**Exp(*B*)**	***B***	**Exp(*B*)**	***B***	**Exp(*B*)**
**Step 1**						
Age	−0.03	−0.04	−0.04	−0.11^**^	0.02	0.05
Gender^a^	1.05	0.05	−0.69	−0.06	0.24	0.02
Group^b^	−5.00	−0.42^***^	−1.44	−0.24^***^	−0.30	−0.05
COVID-19 patient	−2.30	−0.11^**^	–*1.05*	−0.10^**^	–*0.31*	−0.03
Commitment	−0.55	−0.24^***^	−0.24	−0.20^***^	0.12	0.10^*^
Control	−0.09	−0.03	−0.18	−0.12^*^	0.26	0.17^***^
Challenge	0.08	0.03	0.04	0.03	0.35	0.28^***^
	*R*^2^ = 0.320 *F*_(7, 659)_ = 44.392^***^		*R*^2^ = 0.183 *F*_(7, 659)_ = 21.028^***^		*R*^2^ = 0.215 *F*_(7, 659)_ = 25.801^***^	
**Step 2**						
Age	−0.01	−0.01	−0.04	−0.11^**^	0.01	0.03
Gender^a^	0.80	0.04	−0.71	−0.06	0.46	0.04
Group^b^	−4.93	−0.42^***^	−1.46	−0.24^***^	−0.25	−0.04
COVID-19 patient	−2.28	−0.11^**^	−1.05	−0.10^**^	−0.34	−0.03
Commitment	−0.40	−0.17^***^	−0.22	−0.18^**^	0.06	0.05
Control	−0.05	−0.02	−0.17	−0.11^*^	0.21	0.14^**^
Challenge	0.16	0.07	0.06	0.05	0.27	0.21^***^
Problem focused	0.12	0.08	−0.04	−0.05	0.16	0.20^***^
Stop negative T-E	−0.21	−0.21^***^	0.02	0.04	0.01	0.01
Support	−0.07	−0.05	−0.05	−0.07	0.01	0.02
	*R*^2^ = 0.347 Δ*R*^2^ = 0.03*** F_(10, 656)_ = 34.794^***^		*R*^2^ = 0.187 ΔR^2^ = 0.004 *F*_(10, 656)_ = 15.079^***^		*R*^2^ = 0.243 ΔR^2^ = 0.03*** F_(10, 656)_ = 21.037^***^	

*EE, emotional exhaustion; D, depersonalization; PA, personal accomplishment*.

a *Gender (1 = male; 2 = female)*.

b *Group (1 = Health Care worker; 2 = emergency response; 3 = volunteer emergency response)*.

* *p < 0.05*,

** *p < 0.01*,

*** *p < 0.001*.

Hypothesis 5 stipulates that Hardiness and positive coping strategies operate as moderators in the relation between Emergency Stress and Burnout. To test this, total HRG scores, coping strategies, ESQ (total) and Burnout scores were all standardized and entered into several univariate general linear models, along with interaction terms for ESQ ^*^ HRG and for ESQ and each of the three coping scales. Since ESQ showed no effects on Burnout—Personal Accomplishment (PA), no models were tested for PA.

Results (Table 5) showed significant main effects for both Hardiness (HRG) and ESQ on Burnout Emotional Exhaustion (EE) and Depersonalization (D), as well as a significant interaction effect of ESQ ^*^ HRG predicting both of these Burnout components (top of Table 5). Additional significant interaction effects were found for ESQ ^*^ Problem Focused Coping predicting Burnout Depersonalization (D), and for ESQ ^*^ Stopping Negative Thoughts-Emotions predicting Burnout Emotional Exhaustion. Support Coping showed main effects predicting Burnout EE and D, but no interaction effects.

**Table 5:** OLS regression results of ESQ effect on two burnout scales with HRG and coping strategies entered as moderators.

**Parameter**	**EE**			**D**		
	***B***	**Exp (*B*)**	**95% CI**	***B***	**Exp (*B*)**	**95% CI**
Z_ESQ	0.69	24.75^***^	(0.64; 0.75)	0.45	12.95^***^	(0.38; 0.52)
Z_HRG	−0.08	−2.94^**^	(−0.14; −0.03)	−0.15	−4.30^***^	(−0.22; −0.08)
Z_ESQ *Z_HRG	−0.09	−3.68^***^	(−0.14; −0.04)	−0.08	−2.59^*^	(−0.14; −0.02)
Z_ESQ	0.71	25.88^***^	(0.66; 0.77)	0.48	14.04^***^	(0.41; 0.55)
Z_Focus-Problem-Focused	−0.05	−1.63	(−0.10; 0.01)	−0.09	−2.72^**^	(−0.16; −0.03)
Z_ESQ * Z_Problem-Focused	−0.08	−2.83	(−0.13; −0.02)	−0.07	−0.2.14^*^	(−0.14; −0.01)
Z_ESQ	0.68	23.88^***^	(0.62; 0.73)	0.48	0.13.36^***^	(0.41; 0.55)
Z_Stop_Negative T-E	−0.10	−3.41^**^	(−0.16; −0.04)	−0.04	−1.00	(−0.11; 0.04)
Z_ESQ * Z_StopNegativet-E	−0.08	−0.3.10^**^	(−0.13; −0.03)	−0.07	−1.98	(−0.13; −0.00)
Z_ESQ	0.70	25.45^***^	(0.65; 0.75)	0.48	13.91^***^	(0.41; 0.54)
Z_Support	−0.08	−2.87^**^	(−0.14; −0.03)	−0.09	−2.64^**^	(−0.16; −0.02)
Z_ESQ * Z_Support	−0.03	−1.04	(−0.08; 0.03)	−0.05	−1.37	(−0.11; 0.02)

*EE, emotional exhaustion; D, depersonalization*.

* *p < 0.05*;

** *p < 0.01*;

*** *p < 0.001*.

**Figure 1:**
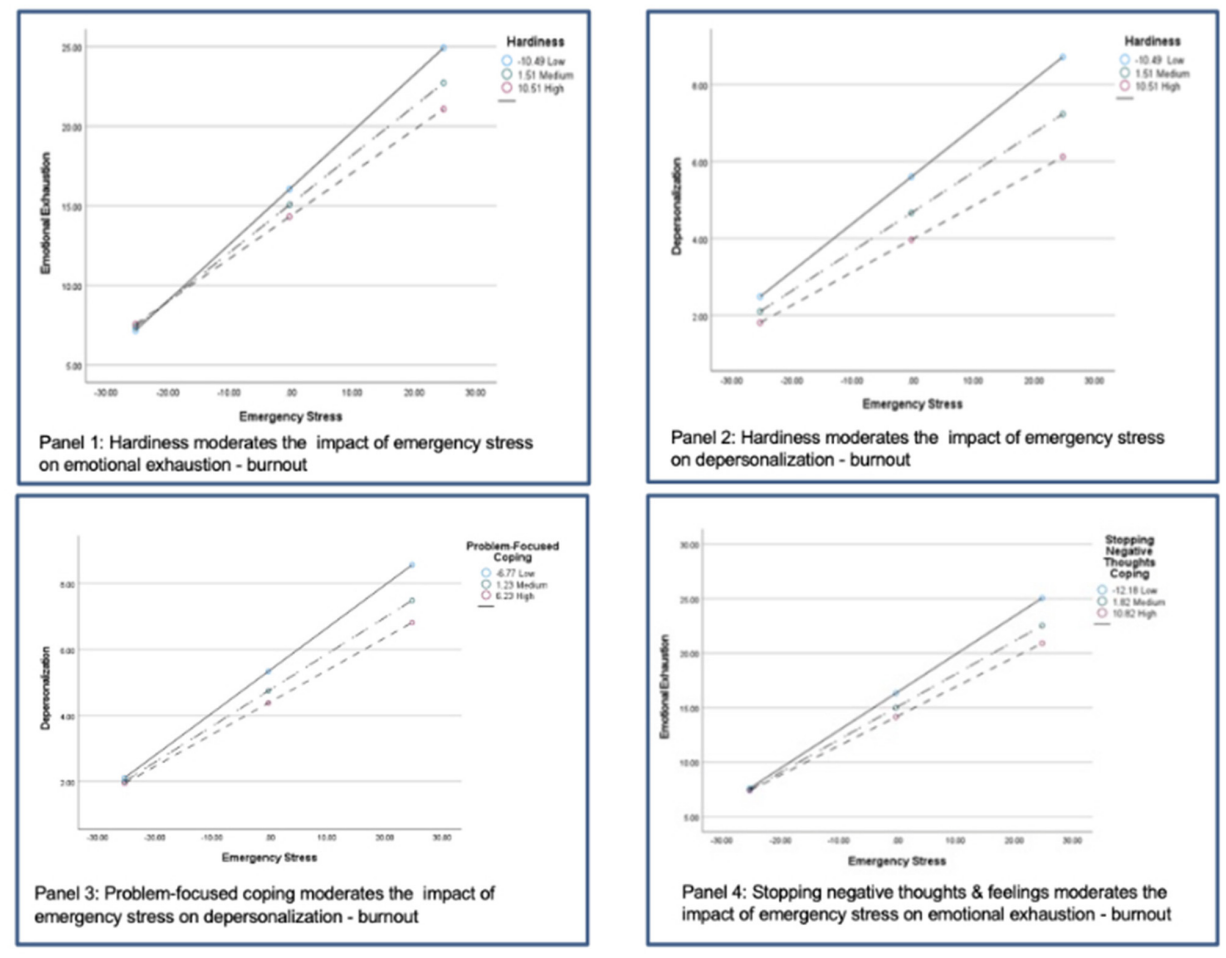
Graphical display of significant interaction effects.

In order to visualize the interactions, significant effects were plotted using the PROCESS (v. 4.0) macro for SPSS (Hayes, 2022). As can be seen in Figure 1, it is at high levels of Emergency Stress that Hardiness has the strongest effects on Emotional Exhaustion (panel 1) and Depersonalization Burnout (panel 2). Subjects low in Hardiness who are experiencing higher levels of stress are also highest in Burnout. Likewise, those low in Problem-Focused coping and high in Emergency Stress are highest in Depersonalization Burnout (panel 3). High levels of Emotional Exhaustion are also seen among the high stress workers who are low in the coping style of Stopping Negative Thoughts-Emotions (panel 4).

## Discussion

In examining Hardiness and coping strategies, we found significant group differences as predicted. The resilience factors of Hardiness and positive coping approaches of focusing on the problem and Stopping Negative Thoughts-Emotions were higher in all three groups of COVID workers (Health Care Workers, Professional Emergency Responders, and Volunteer Emergency Responders) compared to the General Population sample, lending support to Hypothesis 1. One possible explanation is that people who are high in Hardiness and positive coping skills are more attracted to Health Care and emergency service occupations in the first place. Also, the training and mutual support of coworkers and supervisors may play a role in reinforcing positive attitudes and coping skills in these workers (James, 2021). Another likely influence concerns the meaningfulness of the work itself. During the COVID pandemic while much of the population was in lockdown and unable to work or engage in normal activities, Health Care and Emergency Responders were actively engaged in assisting people in need, doing what they were trained and prepared to do. Thus, while these workers are experiencing high levels of work-related stress, at the same time they may benefit by the realization that they are making important contributions to address the crisis, building up a sense of purpose and meaning.

Interestingly, there were no differences between the General Population sample and the COVID worker groups in the coping strategy of finding social support, and all groups were relatively low on this dimension. This makes sense considering that social isolation and social distancing have been medically required in many areas in order to stop the spread of the COVID virus. At the same time, of the various groups examined, it is the Emergency Responders, both volunteer and professional, who report the highest levels of social support. For these workers then, support from coworkers, family and friends appears to be an important stress resistance resource. In contrast, Health Care Workers show significantly lower levels of support compared to Emergency Responders, reflecting their continuing social isolation throughout the pandemic even in the workplace. In this regard, many Health Care Workers have experienced increased social separation and communication difficulties as a result of special precautions and personal protective equipment such as face masks and hoods that must be constantly worn in the workplace. While medically necessary, these measures impose significant communication barriers between the health care workers and their patients, as well as their fellow health care providers.

According to Labrague (2021) Health Care providers during the COVID pandemic were unable to take advantage of social support and used their resilience skills to increase other positive coping strategies. Avoiding persistence in strategies that at that time could not be available is a protective behavior that comes from personal resilience.

In the present study, we included both professional Emergency Responders (firefighters, ambulance medical technicians, and civil protection workers) as well as volunteers (Red Cross workers). Of all the COVID worker groups examined, the Volunteers showed the lowest levels of Burnout. The volunteer workers also showed the highest levels of Hardiness. Other studies have also found greater stress resilience in volunteer workers in high stress jobs. For example, in a study of officers assigned to assist grieving family members after a military death, those who volunteered for this sad duty showed fewer psychiatric symptoms and greater hardiness compared to officers who were ordered to do it (Bartone et al., 1989). It may be that volunteers serving tough duty have a stronger sense of purpose and meaning in their work, which is an important element of the hardiness commitment facet. Also, being a Volunteer Emergency Responder as opposed to a salaried employee may impart a greater sense of control, in that volunteer workers are free to leave at any time without penalty. This expresses Hardiness Control, and at the same time may reinforce it.

Looking at just the General Population sample, Hardiness and positive coping approaches proved predictive of lower stress and fewer stress related problems, supporting Hypothesis 2. Social support did not have an influence here, perhaps again due to restrictions on social activities and contacts during the pandemic. On the other hand, our findings indicate that young people and women are at higher risk for stress reactions. A recent study of burnout in a broad sample of U.S. workers also found that younger workers were at especially high risk (Bartone et al., 2021). Several factors may help to explain this age effect. Younger workers have less job seniority and security than older ones, and so are more vulnerable to layoffs and pay cuts during the pandemic. Also, they generally lack the financial resources and savings needed to make it through hard times. Finally, young people have more limited experience in coping with major social and economic challenges of the sort COVID has brought on, and so are more disrupted by them. A large scale study of the general population in China also found greater risk for psychological problems among young people (Yan et al., 2021). Similar to our own findings, these authors also found women to be at greater risk for COVID stress related problems. One of the reasons cited for this is increased job insecurity and difficulty working experienced by women in the Chinese population. More generally, COVID has led to the loss of many service jobs as in hotels and restaurants, which traditionally employ more women than men. And even when women are able to hold onto their jobs and work from home, they also frequently face the increased burden of child care and education of children who are home all day because schools are closed.

One of the main hypotheses of this research was to examine Emergency Stress and Burnout in Italian workers engaged in the COVID response, while also seeking to identify factors contributing to resilience in these workers. Our findings indicate that Italian Health Care Workers are experiencing the highest levels of Emergency Stress and Burnout, followed by Emergency Responders (Hypothesis 3). These results are in line with other research indicating that Health Care Workers around the world are being most severely affected by this global pandemic (Chutiyami et al., 2022). For example, a review by Spoorthy et al. (2020) found that health care workers in India and China working with COVID patients experienced higher levels of emotional stress, depression, anxiety, depression, and insomnia. Similar findings have been reported with health care workers in Pakistan (Rana et al., 2020), the United Kingdom (Gilleen et al., 2021), and the United States (Prasad et al., 2021). In a 2020 online survey of U.S. health care workers, 76% reported exhaustion and burnout, 86% anxiety, 75% feeling overwhelmed, and 75% with sleep disturbances (Mental Health America, 2022). Recent studies have also found that COVID related stress is linked to a range of psychiatric symptoms as well as intentions to quit the occupation for health care workers and also first responders including firefighters and police (Hendrickson et al., 2022).

Our regression results confirmed that Hardiness and positive coping approaches provide some protection against Burnout (Hypothesis 4), with some different patterns for the Burnout components. Hardiness Commitment shows the largest (negative) effects with Burnout Emotional Exhaustion and Depersonalization. The strong sense of purpose and meaning that comes with Commitment thus appears to protect against these components of Burnout. In contrast, Hardiness Challenge and Control are more strongly (positively) linked to a sense of Personal Accomplishment. Hardiness Challenge entails a readiness to take on new and difficult tasks, as well as accepting disappointments and failures as opportunities to learn and improve. These results are in agreement with Bay and Novinrouz (2022) that demonstrated how being committed to one's activities and believe that life events are challenges for reaching higher professional levels prevent burnout. Moreover, people who have more power to control experience less exhaustion and greater work accomplishment.

For Health Care Workers and Emergency Responders high in Hardiness Challenge, dealing with COVID patients and related problems is apparently a mission they feel is within their skills and capabilities to perform (Control), and an interesting if difficult Challenge to learn from. Problem Focused coping also enters into the model here, suggesting that these workers tend to take action to get things done and solve problems as they come up on the job. Also noteworthy in the regression results, the experience of direct exposure to COVID patients emerged as an additional independent risk factor for developing Burnout Emotional Exhaustion and Depersonalization. This may reflect the workers' ongoing fear of getting infected themselves, or of causing their family members to be infected. Also, their more frequent and close contact with seriously ill and dying COVID patients can lead to increased feelings of sadness and powerlessness.

The coping approach of Stopping Negative Thoughts-Emotions appears to be valuable in protecting against the core Burnout element—Emotional Exhaustion. For workers involved in providing care and support for COVID patients, it is important that they not dwell on negative feelings and ideas as they perform their work. Those who are unable to block out disturbing images and thoughts, for example of sick and dying patients, may experience greater spillover of work into family life, as well as disrupted sleep and eating patterns. This finding is in line with results from a recent study of health care workers in 32 countries, in which maintaining positive thoughts was reported as an important coping mechanism for dealing with COVID related stress (Htay et al., 2021). Related to this, emergency responders including police, firefighters and ambulance workers are known to use dark or “gallows” humor as a way of distancing themselves from traumatic scenes encountered in their work, such as dead, burned and mangled human bodies (Charman, 2013; Dangermond et al., 2022). The use of such humor is a valuable way to replace negative thoughts and emotions with more positive ones, and is associated with better adjustment for emergency service professionals (Rowe and Regehr, 2010).

In some ways the most important question addressed in the present study is if Hardiness or any of the coping approaches interacts with Emergency Stress to reduce or moderate its impact on Burnout (Hypothesis 5). Here, results were somewhat mixed, but overall as predicted. Hardiness is a significant moderator of Emergency Stress on Burnout Emotional Exhaustion and Depersonalization. This is in accord with many previous studies showing that hardiness is a buffer or protective factor in the relation of stress to symptoms and health (Hystad et al., 2009; Eschleman et al., 2010; Johnsen et al., 2017), and in a recent study on hardiness and burnout in U.S. workers (Bartone et al., 2021). Stopping Negative Thoughts and Emotions was a stress moderator with respect to Burnout Emotional Exhaustion. This provides further confirmation that for those on the front lines dealing with the COVID crisis, being able to shift negative thoughts and feelings away and maintain positive thinking is an important coping strategy for fending off the primary element of Burnout: Emotional Exhaustion.

Problem Focused coping also moderates Emergency Stress, but this time with the Depersonalization element of Burnout. Depersonalization or cynicism develops in workers as they become physically and emotionally exhausted, and experience increasing feelings of powerlessness to change the situation or make things better. They slowly become more and more alienated from the job and their coworkers, while also distancing themselves from an unfair system they feel powerless to change. According to our findings, Health Care Workers and Emergency Responders who routinely use Problem Focused coping strategies are more resistant to developing the depersonalization form of Burnout. Problem Focused coping also is quite strongly related to Hardiness in this sample (*r* = 0.58, *p* < 0.001). Health Care and Emergency Workers who are high in hardiness will tend to maintain their commitment and dedication to the work despite the difficulties, keeping their sense of purpose and control.

## Limitations

This study has several limitations that should be mentioned. All data collected were cross-sectional in nature, meaning that definite conclusions regarding causal direction cannot be made. In future studies it would be desirable to collect reports on stress exposure prior in time to Burnout or other outcome measures. It would also be of benefit to have baseline measures of Burnout in order to assess more clearly any COVID stress related increases. The use of self-reporting tools without control scales to detect any response bias such as social desirability can limit the validity of the results. In any case, it should be noted that the results obtained in are in line with other studies in this area.

Despite these limitations, the present research included multiple samples of Health Care Workers, Emergency Responders, and a sample of the General Population for comparison purposes, which provides greater confidence in the validity of our findings. Another potential limitation is that all data were collected in a single country (Italy), and so results may not be fully generalizable to Health Care Workers and Emergency Responders in other countries around the world. However, there is no *a priori* reason to believe that the kinds of COVID related challenges faced by these workers are substantially different from one country to another, at least in western European countries. In underdeveloped countries where medical resources, drugs and equipment are in short supply, if anything the stressors on workers dealing with COVID patients would be even greater, and so Burnout and other ill-effects of stress potentially more severe. This would tend to make protective resources like Hardiness attitudes and coping skills even more important in areas where medical resources are limited.

## Conclusions

This study has demonstrated that Health Care Workers and Emergency Responders are experiencing high levels of stress related to their COVID duties, which is leading to increased Burnout symptoms especially among the Health Care Workers. Volunteer Emergency Responders, as opposed to professionals, are somewhat less vulnerable. Most importantly, Hardiness and positive coping skills provide resistance to the ill-effects of work-related stress in these groups.

While Hardiness and coping strategies are somewhat habitual or trait-like (showing stability over time and across situations), research has shown that specialized training programs can increase both hardiness (Bartone and Homish, 2020; Stein and Bartone, 2020) and positive coping skills (Folkman et al., 1991; Chesney et al., 1996). For example, Judkins et al. (2006) developed a training program to build hardiness in nurses, and MHS Assessments in Canada provides hardiness training and coaching strategies to increase hardiness in a range of occupational settings (Stein and Bartone, 2020; McDonald and Hansma, 2022). Leaders and managers in the health care and emergency responder community may wish to consider incorporating some form of hardiness and positive coping skills training into their existing employee training and support programs.

## Data Availability Statement

The raw data supporting the conclusions of this article will be made available by the authors, without undue reservation.

## Ethics Statement

The studies involving human participants were reviewed and approved by Comitato Etico per la Sperimentazione Umana—CESU of the University of Urbino. The patients/participants provided their written informed consent to participate in this study.

## Author Contributions

MV, TM, VG, DP, and PB: conceptualization, writing—original draft preparation, and writing—review and editing. MV, TM, and VG: methodology and investigation. MV, TM, and PB: formal analysis and data curation. MV and PB: visualization. MV, TM, DP, and PB: project administration. All authors contributed to the article and approved the submitted version.

## Conflict of Interest

The authors declare that the research was conducted in the absence of any commercial or financial relationships that could be construed as a potential conflict of interest.

## Publisher's Note

All claims expressed in this article are solely those of the authors and do not necessarily represent those of their affiliated organizations, or those of the publisher, the editors and the reviewers. Any product that may be evaluated in this article, or claim that may be made by its manufacturer, is not guaranteed or endorsed by the publisher.

## References

[B1] Bartone P. T. Homish G. G. (2020). Influence of hardiness, avoidance coping, and combat exposure on depression in returning war veterans: a moderated-mediation study. J. Affect. Disord. 265, 511–518. 10.1016/j.jad.2020.01.127 32090779

[B2] Bartone P. T. McDonald K. Hansma B. J. (2021). Hardiness and burnout in adult U.S. workers. J. Occup. Environ. Med. 10.1097/JOM.0000000000002448. [Epub ahead of print]. 34817457

[B3] Bartone P. T. McDonald K. Hansma B. J. Stermac-Stein J. Escobar E. M. R. Stein S.. (2022). Development and validation of an improved hardiness measure: the hardiness resilience gauge. Eur. J. Psychol. Assess. 10.1027/1015-5759/a000709. [Epub ahead of print].

[B4] Bartone P. T. Ursano R. J. Wright K. M. Ingraham L. H. (1989). The impact of a military air disaster on the health of assistance workers: a prospective study. J. Nerv. Ment. Dis. 177, 317–328. 10.1097/00005053-198906000-00001 2723619

[B5] Bay F. Novinrouz E. (2022). The predictive effect of early maladaptive schemas and hardiness on burnout of elementary school teachers. Iran. Evol. Educ. Psychol. 4, 73–83. 10.52547/ieepj.4.1.73

[B6] Chan D. W. (2000). Dimensionality of hardiness and its role in the stress-distress relationship among Chinese adolescents in Hong Kong. J. Youth Adolesc. 29, 147–161. 10.1023/A:1005100531194

[B7] Charman S. (2013). Sharing a laugh: the role of humour in relationships between police officers and ambulance staff. Int. J. Sociol. Soc. Policy 33, 152–166. 10.1108/01443331311308212

[B8] Chesney M. Folkman S. Chambers D. (1996). Coping effectiveness training for men living with HIV: preliminary findings. Int. J. STD AIDS 7(2 Suppl.), 75–82. 10.1258/0956462961917690 8799801

[B9] Chesney M. A. Neilands T. B. Chambers D. B. Taylor J. M. Folkman S. (2006). A validity and reliability study of the coping self-efficacy scale. Br. J. Health Psychol. 11, 421–437. 10.1348/135910705X53155 16870053 PMC1602207

[B10] Chutiyami M. Cheong A. Salihu D. Bello U. M. Ndwiga D. Maharaj R.. (2022). COVID-19 pandemic and overall mental health of healthcare professionals globally: a meta-review of systematic reviews. Front. Psychiatry 12:804525. 10.3389/fpsyt.2021.804525 35111089 PMC8801501

[B11] Clarke D. E. (1995). Vulnerability to stress as a function of age, sex, locus of control, hardiness, and type A personality. Soc. Behav. Pers. 23, 285–286. 10.2224/sbp.1995.23.3.285

[B12] Cohen S. Kamarck T. Mermelstein R. (1983). Perceived stress scale (PSS). J. Health Soc. Behav. 24, 385–396. 10.2307/2136404 6668417

[B13] Cohen S. Williamson G. (1988). “Perceived stress in a probability sample of the United States” in The Social Psychology of Health, eds S. Spacapan, and S. Oskamp (Newbury Park, CA: Sage), 31–67.

[B14] Conversano C. Marchi L. Miniati M. (2020). Psychological distress among healthcareprofessionals involved in the COVID-19 emergency: vulnerability and resilience factors. Clin Neuropsychiatry 17, 94. 10.36131/CN20200212 34908976 PMC8629057

[B15] Croghan I. T. Chesak S. S. Adusumalli J. Fischer K. M. Beck E. W. Patel S. R.. (2021). Stress, resilience, and coping of healthcare workers during the COVID-19 pandemic. J. Prim. Care Commun. Health 12, 21501327211008448. 10.1177/21501327211008448 33834900 PMC8040550

[B16] Dangermond K. Weewer R. Duyndam J. Machielse A. (2022). “If it stops, then I'll start worrying.” Humor as part of the fire service culture, specifically as part of coping with critical incidents. HUMOR 35, 31–50. 10.1515/humor-2021-0106

[B17] Dymecka J. Gerymski R. Machnik-Czerwik A. Derbis R. Bidzan M. (2021). Fear of COVID-19 and life satisfaction: the role of the health-related hardiness and sense of coherence. Front. Psychiatry 2, 712103. 10.3389/fpsyt.2021.712103 34790135 PMC8591072

[B18] Eschleman K. J. Bowling N. A. Alarcon G. M. (2010). A meta-analytic examination of hardiness. Int. J. Stress Manag. 17, 277–307. 10.1037/a0020476

[B19] Folkman S. Chesney M. McKusick L. Ironson G. Johnson D. S. Coates T. J. (1991). “Translating coping theory into an intervention” in The Social Context of Coping. The Springer series on stress and coping, ed J. Eckenrode (Boston, MA: Springer).

[B20] Gilleen J. Santaolalla A. Valdearenas L. Salice C. Fusté M. (2021). Impact of the COVID-19 pandemic on the mental health and well-being of UK healthcare workers. BJPsych Open 7, E88. 10.1192/bjo.2021.42 33910674 PMC8082128

[B21] Hayes A. F. (2022). Introduction to Mediation, Moderation, and Conditional Process Analysis: A Regression Based Approach, 3rd Edn. New York, NY: Guilford.

[B22] Hendrickson R. C. Slevin R. A. Hoerster K. D. Chang B. P. Sano E. McCall C. A.. (2022). The impact of the COVID-19 pandemic on mental health, occupational functioning, and professional retention among health care workers and first responders. J. Gen. Intern. Med. 37, 397–408. 10.1007/s11606-021-07252-z 34918181 PMC8675543

[B23] Hines S. E. Chin K. H. Glick D. R. Wickwire E. M. (2021). Trends in moral injury, distress, and resilience factors among healthcare workers at the beginning of the COVID-19 pandemic. Int. J. Environ. Res. Public Health 18, 488. 10.3390/ijerph18020488 33435300 PMC7826570

[B24] Htay M. Marzo R. R. Bahari R. AlRifai A. Kamberi F. El-Abasiri R. A.. (2021). How healthcare workers are coping with mental health challenges during COVID-19 pandemic? a cross-sectional multi-countries study. Clin. Epidemiol. Glob. Health 11, 100759. 10.1016/j.cegh.2021.100759 33977169 PMC8103713

[B25] Hystad S. W. Eid J. Laberg J. C. Johnsen B. H. Bartone P. T. (2009). Academic stress and health: exploring the moderating role of personality hardiness. Scand. J. Educ. Res. 53, 421–429. 10.1080/00313830903180349

[B26] James D. (2021). Hardiness and attitudes toward professional Health Care services: implications for Health Care service utilization among Black American adults. Health Psychol. Open 8(2), 20551029211029157. 10.1177/20551029211029157 34377525 PMC8323433

[B27] Johnsen B. H. Espevik R. Saus E. R. Sanden S. Olsen O. K. Hystad S. W. (2017). Hardiness as a moderator and motivation for operational duties as mediator: the relation between operational self-efficacy, performance satisfaction, and perceived strain in a simulated police training scenario. J. Police Crim. Psychol. 32, 331–339. 10.1007/s11896-017-9225-1

[B28] Judkins S. Reid B. Furlow L. (2006). Hardiness training among nurse managers: building a healthy workplace. J. Contin. Educ. Nurs. 37, 202–238. 10.3928/00220124-20060901-03 17004392

[B29] Kamtsios S. Bartone P. (2021). Preliminary evaluation of the psychometric properties of the “Hardiness-Resilience Gauge” in an undergraduate sample. Hell. J. Psychol. 18, 287–310. 10.26262/hjp.v18i3.8205

[B30] Kobasa S. C. (1979). Stressful life events, personality, and health: an inquiry into hardiness. J. Pers. Soc. Psychol. 37, 1–11. 10.1037//0022-3514.37.1.1 458548

[B31] Kobasa S. C. (1982). Commitment and coping in stress resistance among lawyers. J. Pers. Soc. Psychol. 42, 707–717. 10.1037/0022-3514.42.4.707

[B32] Kobasa S. C. Maddi S. R. Kahn S. (1982). Hardiness and health: a prospective study. J. Pers. Soc. Psychol. 42, 168–177. 10.1037/0022-3514.42.1.168 7057354

[B33] Labrague L. J. (2021). Psychological resilience, coping behaviours and social support among health care workers during the COVID-19 pandemic: a systematic review of quantitative studies. J. Nurs. Manag. 29, 1893–1905. 10.1111/jonm.13336 33843087 PMC8250179

[B34] Lease S. (1999). Occupational role stressors, coping support and hardiness as predictors of strain in academic faculty: an emphasis on new and female faculty. Res. High Educ. 40, 285–307. 10.1023/A:1018747000082

[B35] Loera B. Converso D. Viotti S. (2014). Evaluating the psychometric properties of the Maslach Burnout Inventory–Human Service Survey (MBI-HSS) among Italian nurses: how many factors must a research consider? PLoS ONE 9:e114987. 10.1371/journal.pone.0114987 25501716 PMC4264862

[B36] Maiorano T. Vagni M. Giostra V. Pajardi D. (2020). COVID-19: risk factors and protective role of resilience and coping strategies for and secondary trauma in medical staff and emergency workers—an online-based inquiry. Sustainability 12, 9004. 10.3390/su12219004

[B37] Manchia M. Gathier A. W. Yapici-Eser H. Schmidt M. V. de Quervain D. van Amelsvoort T.. (2022). The impact of the prolonged COVID-19 pandemic on stress resilience and mental health: a critical review across waves. Eur. Neuropsychopharmacol. 55, 22–83. 10.1016/j.euroneuro.2021.10.864 34818601 PMC8554139

[B38] Maslach C. Jackson S. E. (1981). The measurement of experienced burnout. J. Organiz. Behav. 2, 99–113. 10.1002/job.4030020205

[B39] Maslach C. Jackson S. E. (1986). Maslach Burnout Inventory, 2nd Edn. Palo Alto, CA: Consulting Psychologists Press.

[B40] Maslach C. Jackson S. E. Leiter M. P. (1996). MBI: Maslach Burnout Inventory, 3rd Edn. Sunnyvale, CA: Consulting Psychologists Press.

[B41] McDonald K. Hansma B. (2022). Harness Hardiness to Protect Yourself and Your Team Against Burnout. MHS Talent Assessment blog post. Available online at: https://mhs.com/harness-hardiness-to-protect-yourself-and-your-team-against-burnout/ (accessed March 16, 2022).

[B42] Mental Health America (2022). The Mental Health of Healthcare Workers in COVID-19. Available online at: https://mhanational.org/mental-health-healthcare-workers-covid-19#:~:text=The%20responses%20collected%20from%20the,75%25%20said%20they%20were%20overwhelmed (accessed March 17, 2022)

[B43] Prasad K. McLoughlin C. Stillman M. Poplau S. Goelz E. Taylor S.. (2021). Prevalence and correlates of stress and burnout among US healthcare workers during the COVID-19 pandemic: a national cross-sectional survey study. eClinicalMedicine 35, 100879. 10.1016/j.eclinm.2021.100879 34041456 PMC8141518

[B44] Rana W. Mukhtar S. Mukhtar S. (2020). Mental health of medical workers in Pakistan during the pandemic COVID-19 outbreak. Asian J. Psychiatr. 51, 102080. 10.1016/j.ajp.2020.102080 32283512 PMC7139243

[B45] Rowe A. Regehr C. (2010). Whatever gets you through today: an examination of cynical humor among emergency service professionals. J. Loss Trauma 15, 448–464. 10.1080/15325024.2010.507661

[B46] Sirigatti S. Stefanile C. (1993). Adattamento Italiano del MBI-Maslach Burnout Inventory. Firenze: Giunti Organizzazioni Speciali.

[B47] Spoorthy M. S. Pratapa S. K. Mahant S. (2020). Mental health problems faced by healthcare workers due to the COVID-19 pandemic—a review. Asian J. Pychiatr. 51, 102119. 10.1016/j.ajp.2020.102119 PMC7175897 32339895

[B48] Stein S. J. Bartone P. T. (2020). Hardiness: Making Stress Work for You to Achieve Your Life Goals. Hoboken, NJ: Wiley.

[B49] Thomassen Å. G. Hystad S. W. Johnsen B. H. Johnsen G. E. Bartone P. T. (2018). The effect of hardiness on PTSD symptoms: a prospective mediational approach. Mil. Psychol. 30, 142–151. 10.1080/08995605.2018.1425065

[B50] Thomassen Å. G. Johnsen B. H. Hystad S. W. Johnsen G. E. (2022). Avoidance coping mediates the effect of hardiness on mental distress symptoms for both male and female subjects. Scand. J. Psychol. 63, 39–46. 10.1111/sjop.12782 34676897

[B51] Vagni M. Giostra V. Maiorano T. Santaniello G. Pajardi D. (2020a). Personal accomplishment and hardiness in reducing emergency stress and burnout among COVID-19 emergency workers. Sustainability 12, 9071. 10.3390/su12219071

[B52] Vagni M. Maiorano T. Giostra V. Pajardi D. (2020b). Coping with COVID-19: emergency stress, secondary trauma and self-efficacy in healthcare and emergency workers in Italy. Front. Psychol. 11, 566912. 10.3389/fpsyg.2020.566912 33013603 PMC7494735

[B53] Vagni M. Maiorano T. Giostra V. Pajardi D. (2020c). Hardiness and coping strategies as mediators of stress and secondary trauma in emergency workers during the COVID-19 pandemic. Sustainability 12, 7561. 10.3390/su12187561

[B54] Vagni M. Maiorano T. Giostra V. Pajardi D. (2020d). Hardiness, stress and secondary trauma in italian healthcare and emergency workers during the COVID-19 pandemic. Sustainability 12, 5592. 10.3390/su1214559235162544

[B55] Vagni M. Maiorano T. Giostra V. Pajardi D. (2021). Protective factors against emergency stress and burnout in healthcare and emergency workers during second wave of COVID-19. Soc. Sci. 10, 178. 10.3390/socsci10050178

[B56] White A. Zapata I. Lenz A. Ryznar R. Nevins N. Hoang T. N.. (2020). Medical students immersed in a hyper-realistic surgical training environment leads to improved measures of emotional resiliency by both hardiness and emotional intelligence evaluation. Front. Psychol. 11, 569035. 10.3389/fpsyg.2020.569035 33329208 PMC7714941

[B57] Yan S. Xu R. Stratton T. D. Kavcic V. Luo D. Hou F.. (2021). Sex differences and psychological stress: responses to the COVID-19 pandemic in China. BMC Public Health 21, 79. 10.1186/s12889-020-10085-w 33413224 PMC7789895

